# Comments on article by Pullakhandam et al: Reference cut-offs to define low serum zinc concentrations in healthy 1-19 year old Indian children and adolescents

**DOI:** 10.1038/s41430-022-01174-7

**Published:** 2022-07-08

**Authors:** Kenneth H. Brown, Reed Atkin, Jonathan Gorstein, Saskia J. M. Osendarp

**Affiliations:** 1 Department of Nutrition and Institute for Global Nutrition, University of California Davis, Davis, CA USA; 2 The Micronutrient Forum, Washington DC, USA; 3 The Bill & Melinda Gates Foundation, Seattle, WA USA; 4 Department of Global Health, University of Washington, Seattle, WA USA

**Keywords:** Biomarkers, Biological techniques

## To the Editor**:**

Serum zinc concentration (SZC) is generally accepted as the best indicator of population zinc status [1], but there is limited information on SZC worldwide [2]. Pullakhandam et al. [3] have previously reported SZC results from the Indian Comprehensive National Nutrition Survey (ICNNS) conducted from 2016 to 2018 [4], which indicated a high prevalence of zinc deficiency among children and adolescents, based on SZC cutoffs proposed by the International Zinc Nutrition Consultative Group (IZiNCG) [5]. These “IZiNCG cutoffs” were derived from the distribution of SZC values observed among a sample of presumably healthy US children assessed in 1976–80 [6] and largely confirmed in a subsequent survey [7]. As noted previously [8], the findings from the ICNNS seem plausible, and they warrant consideration of public health interventions to reduce the risk of zinc deficiency. However, in a recent article published in the European Journal of Clinical Nutrition [9], Pullakhandam et al. reanalyzed the ICNNS data using lower SZC cutoffs, based on the distribution of values among a presumably healthy subset of individuals assessed in the same national survey, and concluded that zinc deficiency is not a serious public health problem in India. These apparently contradictory results highlight several issues regarding appropriate biomarker cutoffs for identifying zinc and other micronutrient deficiencies.

Biomarker cutoffs used to indicate a nutrient deficiency can be established using two different conceptual approaches. Ideally, these cutoffs are based on the level of the marker at which clinical signs of disease or functional or metabolic disorders begin to appear. However, in situations where such relationships have not been unequivocally established, an alternative approach is to examine the distribution of the biomarker values in presumably healthy, non-malnourished populations and apply a statistical definition (usually the 2.5 percentile) to distinguish between “normal status” and an increased risk of deficiency. The latter approach is less suitable for two possible reasons. First, the reference population might have considerably higher status than needed to prevent adverse outcomes, so the cutoff could be higher than necessary. Second, the reference population may not in fact be healthy, which could yield a lower cutoff than desirable.

In the case of SZC, Wessells et al. found a clear relationship between SZC and the presence of clinical signs of zinc deficiency, both in adult volunteers exposed to experimental zinc deficiency and in patients with acrodermatitis enterpathica [10]. However, as often occurs with nutritional biomarkers, there were overlapping distributions of SZC among those with and without deficiency signs, so no single cutoff provided perfect discrimination. In such cases, establishing a cutoff requires a tradeoff between sensitivity and specificity and judgement regarding which is more important in a particular situation. Such decisions may involve consideration of 1) the related disease severity, which if severe would call for applying greater sensitivity (higher cutoff, fewer false negatives), and 2) the cost of interventions relative to available resources and any potential adverse effects of these interventions, which would argue for greater specificity (lower cutoff, fewer false positives). In the case of zinc deficiency, where the consequences may be severe [11, 12], greater sensitivity seems preferable to allow for intervention before clinical deficiency signs become apparent. Nevertheless, individual countries might choose to apply a lower cutoff if they are willing to tolerate a greater risk of deficiency or available resources only permit limited intervention.

The use of different country-specific cutoffs poses a dilemma with regard to tracking deficiency prevalence globally and related resource allocations. One possible solution would be to agree on a single global cutoff for tracking purposes, while individual countries could determine local cutoffs to trigger programmatic responses. In cases where the cutoff is based on statistical criteria, the reference population must be healthy, adequately nourished, and ideally representative of the global population, not just a single country.

Addressing this set of issues will require global consensus on best practices to develop appropriate biomarker cutoffs to define MN deficiency (and excess) and efforts to compile or collect relevant data. The Micronutrient Forum has recently established the Data Innovation Alliance (DInA) to facilitate this process. DInA is engaging micronutrient data users and producers at global and national levels to generate consensus recommendations to ensure that micronutrient data are both consistent globally and relevant for national decision makers.

In summary, the Indian nutrition and public health communities should be applauded for generating data on the population’s zinc status. As new data become available, they should be reported according to international consensus criteria; but the interpretation of these results and related policymaking are the responsibility and prerogative of national stakeholders.
